# The challenge of treating hepatitis C virus-associated cryoglobulinemic vasculitis in the era of anti-CD20 monoclonal antibodies and direct antiviral agents

**DOI:** 10.18632/oncotarget.16986

**Published:** 2017-04-09

**Authors:** Dario Roccatello, Savino Sciascia, Daniela Rossi, Laura Solfietti, Roberta Fenoglio, Elisa Menegatti, Simone Baldovino

**Affiliations:** ^1^ Department of Clinical and Biological Sciences, Center of Research of Immunopathology and Rare Diseases, Coordinating Center of the Network for Rare Diseases of Piedmont and Aosta Valley, S. Giovanni Bosco Hospital and University of Turin, Turin, Italy; ^2^ Nephrology and Dialysis Unit, S. Giovanni Bosco Hospital and University of Turin, Turin, Italy

**Keywords:** mixed cryoglobulinemia, HCV-associated cryoglobulinemic vasculitis, HCV associated membranoproliferative glomerulonephritis, polyneuropathies, necrotizing skin ulcers

## Abstract

Mixed cryoglobulinemia syndrome (MC) is a systemic vasculitis involving kidneys, joints, skin, and peripheral nerves. While many autoimmune, lymphoproliferative, and neoplastic disorders have been associated with this disorder, hepatitis C virus (HCV) is known to be the etiologic agent in the majority of patients. Therefore, clinical research has focused on anti-viral drugs and, more recently, on the new, highly potent Direct-acting Antiviral Agents (DAAs). These drugs assure sustained virologic response (SVR) rates >90%. Nevertheless, data on their efficacy in patients with HCV-associated cryoglobulinemic vasculitis are disappointing, possibly due to the inability of the drugs to suppress the immune-mediated process once it has been triggered.

Despite the potential risk of exacerbation of the infection, immunosuppression has traditionally been regarded as the first-line intervention in cryoglobulinemic vasculitis, especially if renal involvement is severe. Biologic agents have raised hopes for more manageable therapeutic approaches, and Rituximab (RTX), an anti CD20 monoclonal antibody, is the most widely used biologic drug. It has proved to be safer than conventional immunosuppressants, thus substantially changing the natural history of HCV-associated cryoglobulinemic vasculitis by providing long-term remission, especially with intensive regimens.

The present review focuses on the new therapeutic opportunities offered by the combination of biological drugs, mainly Rituximab, with DAAs.

## INTRODUCTION

Mixed cryoglobulinemia syndrome (MC) is an idiopathic or secondary vasculitis characterised by the presence of mixed cryoglobulins in the circulation, and deposition in target organs. MC may be associated with several conditions including chronic infections, lymphoproliferative disorders, connective tissue diseases, or non-infectious hepatobiliary conditions. Mixed cryoglobulins are cryoprecipitable immune complexes consisting either of a monoclonal immunoglobulin (Ig), usually an IgM-k, plus a polyclonal Ig-k or lambda (type II cryoglobulins), or two polyclonal Igs (type III). In a considerable number of patients previously classified as type III the more advanced immunofixation techniques allow recognition of an intermediate clonal restriction in which an oligoclonal Ig is complexed with a polyclonal Ig type (type II/III cryoglobulins) [1].

Most of the cases that were previously described as “idiopathic or essential” are now associated with the presence of hepatitis C virus (HCV) infection [2–7]. Circulating (usually asymptomatic) cryoglobulins are detectable in up to 40% of patients with chronic hepatitis C. A specific cryoglobulinemic syndrome occurs in a minority of cases (1-2%) [1]. Long-term HCV infection, older age and genetic background all represent predisposing factors for the development of MC [1,8,9].

Renal manifestations involve only 0.1-0.2% of HCV-infected, but the presence of glomerulonephritis is a major long-term prognostic factor for MC.

## RELEVANT PATHOGENIC ASPECTS INVOLVED IN THE CHOICE OF THERAPY

As well as infecting hepatocytes, HCV also infects B-lymphocytes, macrophages, peripheral dendritic cells and monocytes [10]. More specifically, HCV-RNA, HCV NS3 and core proteins have been restrictively found in CD19-positive B cells [11]. HCV-persistent viral stimulation enhances the expression of lymphomagenesis-related genes and may induce a poly- and subsequently monoclonal expansion of B-cells [12]. Under this trigger effect, permanent clones of B-lymphocytes produce oligo- or monoclonal IgM that are known to display rheumatoid factor activity, favouring the formation of immune-complexes (ICs) formed by the monoclonal IgM itself, HCV, and anti-HCV polyclonal IgG antibodies. The erythrocyte transport system does not recognize the cryoprecipitable ICs because of the clonally restricted IgM [13]. Therefore, these ICs escape the splenic and hepatic macrophage removal system [14]. Similarly, monocytes accumulating in the glomeruli of patients with MC are not able to process internalised ICs, thus potentially perpetuating glomerular damage [14]. In a murine model of MC-membranoproliferative glomerulonephritis, macrophage depletion protects animals from glomerulonephritis without affecting cryoglobulin removal [15]. This is consonant to the observation that macrophage-driving mesangial expansion and activation are sustained by the release of pro-inflammatory cytokines and pro-cathepsin D from damage-associated molecular patterns-activated macrophages (Table 1) [16].

**Table 1:** Pathogenetic scenario of cryoglobulinemic nephritis (Ref #42)

Pathogenetic scenario of cryoglobulinemic nephritis
Chronic stimulation by HCV infection sustaining the synthesis of IgM rheumatoid factor (and consequently of cryoprecipitable ICs)
Abnormal kinetics and tissue deposition of the HCV-containing ICs
Ineffective cryoglobulin clearance by monocyte/macrophages, which are implicated in perpetuating glomerular damage.

Abbreviations are: ICs, immune complexes

Based on these pathogenic principles it seems unlikely that pure antiviral agents can effectively interfere with the pathogenesis of MC vasculitis and impact the development of the immune-mediated injury once the immune process is definitively triggered.

### MC-related glomerulonephritis

Table 2 summarises clinical presentations. Renal biopsy should be performed in any patient with urinary abnormalities, unexplained renal impairment or both. Quality and degree of the histological features, which do affect therapy, are clinically unpredictable making examination of renal specimens by light microscopy, immunofluorescence and electron microscopy mandatory.

**Table 2:** Clinical presentations of patients with cryoglobulinemic glomerulonephritis (Ref #7)

Clinical presentations of patients with cryoglobulinemic glomerulonephritis
Isolated proteinuria (<3 g/24 h), usually with microscopic hematuria (30%)
Nephrotic syndrome (20%)
Acute nephritic syndrome (15%). Some patients present with a mixed nephrotic and nephritic syndrome.
Macroscopic hematuria (10%)
Chronic renal insufficiency (10%)
Acute renal failure (10%)
Oligoanuria (5%)

**Figure 1:**
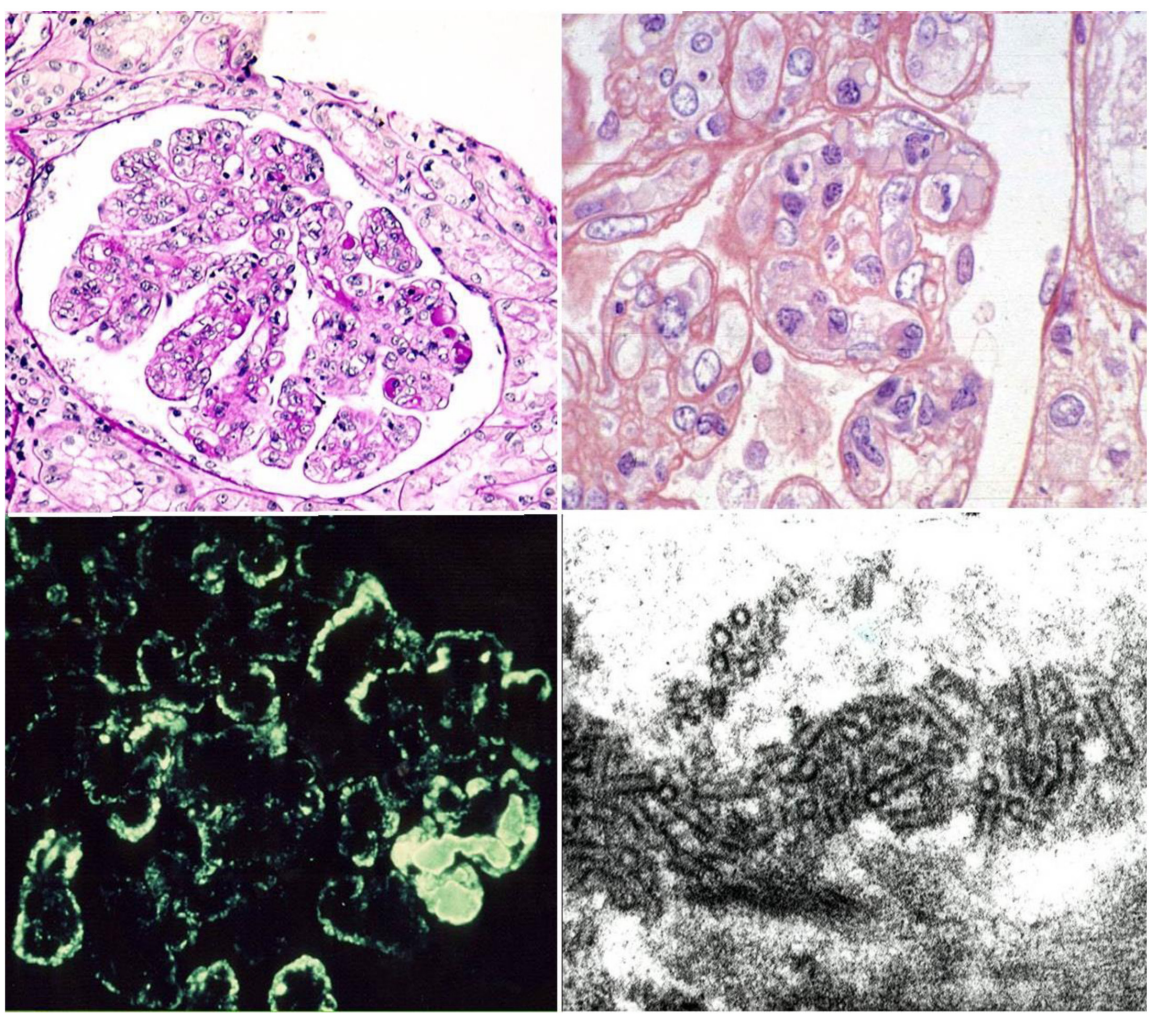
This picture shows the main features of cryoglobulinemic nephritis Light microscopy: Upper left side membranoproliferative pattern. Many loops contain pale eosinophilic material consistent with cryoglobulins. Upper right side higher power magnification showing double contour formation. Immunofluorescence (lower left side): subendothelial and mesangial deposition of immune reactants. Electron Microscopy (lower right side): structured appearance of electron dense deposits.

Analysis by light microscopy allows the identification of three main glomerular patterns of cryoglobulin glomerular deposition (Figure 1) (summarised in [17]).

Diffuse Membranoproliferative Glomerulonephritis (GN) (seen in up to 80% of patients). Typical immunohistological findings include >1. duplication of glomerular basement membrane, 2. interposition by mesangial cells and mainly mononuclear leukocytes/macrophages, 3. subendothelial and mesangial deposition of immune reactant, 4. proliferation and expansion of the mesangium and intracapillary leukocyte accumulation, with endoluminal hyaline pseudothrombi (corresponding to cryoglobulin precipitates). More than 50 % of glomeruli are usually affected. Extracapillary proliferation and necrosis of the glomerular tuft may occasionally be observed.

Focal Membranoproliferative Glomerulonephritis (seen in up to 10% of patients). Immunohistological findings are similar to the previously described features, but are found in less than 50 % of glomeruli. Endoluminal thrombi are rarely seen.

Mesangial Proliferative Glomerulonephritis (10% of patients). Typical immunohistological findings include diffuse mesangial expansion and proliferation in the absence of endocapillary proliferation or exudation. Endoluminal thrombi are rare.

Immunofluorescence staining shows diffuse pseudo-linear mesangial deposition of IgM, IgG, and C3, with a relatively stronger staining for IgM and k (as compared to lambda) light chain (Figure 1) affecting the peripheral capillary wall. Strong IgM and IgG deposition is observed in the thrombi, while fibrinogen can be detected in the vessel walls as a result of a vasculitic process.

Electron-dense deposits can be detected by electron microscopy in the subendothelial and mesangial areas along with interposition of the glomerular basement membrane by monocytes. Cryoglobulin deposits are organised in curved, short, thick-walled tubular structures (diameter of about 30 nm) which appear circular on cross sections (Figure 1).

It must be emphasised that the same clinical weight cannot be assigned to all histological patterns. Thus, the results of clinical studies which are not based on a careful histological examination should be evaluated with caution.

## PROGNOSTIC FACTORS

Cryoglobulinemic vasculitis bears significant morbidity and mortality. Renal involvement has traditionally been considered one of the worst prognostic factor [18]. More recently, Terrier and co-workers showed that the 1-year, 3-year, 5-year, and 10-year survival rates in HCV-positive patients with MC-vasculitis were 96%, 86%, 75%, and 63%, respectively [19]. Severe infections and end-stage liver disease were the main determinants of fatal events. Baseline parameters associated with a poor prognosis included the presence of severe liver fibrosis (Metavir fibrosis score ≥ 3; hazard ratio [HR] 5.31), heart involvement (HR 4.2), CNS involvement (HR 2.74), and renal involvement (HR 1.91) [19]. The Five-Factor Score (FFS) [20], a vasculitis scoring system based on 5 clinical variables (serum creatinine>140 μmol/L, proteinuria > 1 g/d, cardiomyopathy, severe gastrointestinal involvement, and central nervous system involvement), was used as parameter of poor outcome, and results were confirmed by multivariate analysis [20]. Similarly, severe fibrosis (HR 10.8) negatively impacted overall survival [20]. In a large, multicenter study on HCV-related MC, our group demonstrated that significant prognostic variables affecting the outcome of therapy included male gender, age, proteinuria and creatinine assessed at renal biopsy, the number of relapses, and poor blood pressure control [1]. Basal creatinine values >1.5 mg/dL (133 μmol/L) significantly impacted on survival as shown by Kaplan-Meier curves analysis. Cardiovascular disease was found to be the cause of death in over 60% of cases [1].

In a survey from the French CryoVas study, Terrier at al. [21] observed that purpura [HR 3.35 (1.02-10.97)], cutaneous necrosis [HR 4.46 (1.58-12.57)] and articular involvement [HR 2.20 (1.00-4.78)] were significantly associated with early relapse.

### Conventional treatment of cryoglobulinemic vasculitis

Although the viral aetiology of MC is now unquestionable, immunosuppression is still considered the first-line therapeutic approach in MC vasculitis, particularly when kidney involvement is severe [21]. In fact, antiviral treatment often lacks efficacy and might risk being harmful in patients with severe renal involvement [17]. Many centres, despite the absence of properly designed prospective trials, treat patients with plasmapheresis, high-dose glucocorticoids, and cytotoxic agents for the management of more severe cases. These strategies may potentially lead to an increase in viremia and an exacerbation of chronic HCV hepatitis. However, kidney involvement is often a critical indication for these approaches. Another indication remains peripheral neuropathy although it is known to be refractory to treatment in severe cases. In clinical practice, oral (1.5-2 mg/kg/day for 3 months), or intravenous (0.5-1 g every 2-4 weeks) cyclophosphamide, together with oral glucocorticoids (0.5-1 mg/kg/day for 30 days, then tapered by 2.5-5 mg/week), usually preceded by intravenous methylprednisolone (e.g. 3 infusions of 10-15 mg/kg) is the most common regimen for severe manifestations of MC [22]. An alternative immunosuppressive choice may be the administration of mycophenolate mofetil (a less toxic medication) for 6 months. Cyclosporine is known to inhibit the polymerase binding to viral RNA [23] and to significantly reduce viremia within months.

Plasma exchange, mainly double-filtration plasmapheresis, is still being used successfully in escalation protocols for the managements of the more severe manifestations of MC (hyperviscosity syndrome, glomerulonephritis, cutaneous ulcers, systemic vasculitis, and mixed neuropathy). Carrying out an aphaeresis every other day for fifteen days followed by one procedures/ a week for 2 weeks and one procedure a week for a month is the currently recommended schedule [24].

### Mixed cryoglobulinemia as a unique pathologic condition supporting the therapeutic use of the anti-CD20 monoclonal antibody Rituximab

Of the many biologic agents that have shown promising results in the management of patients with severe vasculitis Rituximab (RTX) is the one that has been used extensively to treat mixed cryoglobulinemia [25–40].

Rituximab is a humanised chimeric mouse monoclonal antibody directed at the lymphocyte membrane protein CD20, and it selectively depletes the CD20+ B lymphocytes, thereby abolishing IgM production and new cryoglobulin formation. It is noteworthy that HCV proteins and RNA have been restrictively detected in these cells [11]. Presently, more than 400 Rituximab-treated patients with mixed cryoglobulinemia have been reported in the literature. Table 3 summarises the published literature regarding the use of Rituximab in MC. Briefly, Rituximab has been efficacious in the management of the clinical manifestations of MC. Of note, it improved skin ulcers, renal manifestations in 75-90% of cases (usually within three months), and sensitive-motor neuropathy in 70% of cases. Our group also showed that mixed peripheral neuropathy improved within 5 months after administering Rituximab [32]. Moreover, Rituximab treatment has been found to deplete bone marrow B-cell clonal expansion resulting in a decrease of serum cryoglobulins and rheumatoid factor, and in a normalisation of C4 levels [41–45].

**Table 3:** Study characteristics and clinical outcome of rituximab-treated patients with mixed cryoglobulinemia (MC) reported in the literature

				MC classification		Rituximab		Outcome CR/PR/NR(%)	Renal Involvement (N.)	Biopsy Proven (Y/N)	Renal Outcome* CR/PR/NR (%)	Flare-related re-treament	Adverse Effects
Author (Year)	Pts N.	Study	Follow-up (months)	Essential	HCV+	Protocol	Main indication						
Sansonno (2003)	20	PCS	12	-	20	375mg/m^2^ x 4 weekly	MC-Overall Skin Vasculitis 16 Skin Ulcers 7 Neuropathy 12	80/0/20 75/12/12 42/29/29 50/40/10	1	N	0/0/100	NR	3 Mild
Zaja (2003)	15	PCS	9-31	3	12	375mg/m^2^ x 4 weekly	Skin Vasculitis 12 Neuropathy 7 B-NHL 3 Skin Ulcers 5	75//8/17 86/14/0 33/64/0 100/0/0	2	Y	50/0/50	NR	1 retinal artery thrombosis and 2 panniculitis
De Vita (2007)	28	PCS	3 to 60	NA	NA	375 mg/m 2 weekly for 4 weeks (21/28) or 1 g x 2 (7/28) administered on days 1 and 15.	Skin Vasculitis19 skin ulcers8 neuropathy 15	68/NR/NR 63/NR/NR 86/NR/NR	8	Y	37/NR/NR at month +6	9	3 first infusion reactions, 1 retinal artery thrombosis, 1panniculitis, 1severe infection, 1severe transient neutropenia, 1haemorrhagic alveolitis, 2transaminase elevations
Saadoun (2008)	16	PCS	mean 19.4 (SD 3.6)	-	16	375mg/m^2^ x 4 weekly+peg-IFN-α and RBV	MC-Overall Neuropathy 13 B-NHL 3 Skin Ulcers 2	32/31/6 77/15/8 67/33/0 100/0/0	7	N	57/0/43	NR	12 mild
Roccatello (2008)	12	PCS	24	1	11	375mg/m^2^ x 4 weekly+2 monthly infusions	Skin Ulcer 3 bone marrow clonal restriction 3	100/0/0 100/0/0	7	Y	80/20/0	NR	None
Sène (2009)	22	CS	-	-	22	375mg/m^2^ x 4 weekly (18 patients) 1 g x 2 administered on days 1 and 15 (4 patients)	Purpura 17 polyneuropathy 19	NR	10	Y	NR	NR	27.3% infusion relatedadversereactions. 4 severe flare of MC vasculitis 2 serum sickness syndrome
Cavallo (2009)	13	PCS	12	1	12	375mg/m^2^ x 4 weekly+2 monthly infusions	Neuropathy	Asthenia 83/17/0 Pareshesia50/25/25 Burning feet 67/33/0	None	N/A	NR	NR	NR
Terrier (2009)	32	PCS	24	-	32	375mg/m^2^ x 4 weekly+peg-IFN-α and RBV (20 patients) and 375mg/m^2^ x 4 weekly alone (12)	Overall Overall	80/15/5 58/9/33	NR	N	NR	15% 33%	serum sickness (n = 6), neutropenia (n = 2), varicella zoster virus infection (n = 1), and subcutaneous extravasation of rituximab (n = 1).
Saadoun (2010)	39	PCS	6.8 (SD 4.7)	-	39	375mg/m^2^ x 4 weekly+peg-IFN-α and RBV	Overall Purpura 29 polyneuropathy 28 arthralgia 15	73/23/4 83/0/17 50/0/50	21	N	80/NR/NR	RTX in 2 and peg-IFN-α and RBV in 1	included serum sickness in 4, neutropenia and 2 *Streptococcus* *pneumoniae*pneumopathy, varicella-zoster virus infection, anderysipela (n =1)
Terrier (2010)	23	PCS	22.2 (SD16.7)	8	0	375mg/m^2^ x 4 weekly (18 patients) 1 g x 2 administered on days 1 and 15 (3 patients) R-CHOP (1) Fludarabin+Cylophosphamide (1)	Skin Vasculits 19 Neuropathy 12	74//16/0 84/16/0	7	N	57/13/30	9	Severe infections 6 (3 death), mild adverse events: 2, serum sickness-like syndrome 1
Dammacco (2010)	37	RCT	36	-	37	375mg/m^2^ x 4 weeklyfollowed by two 5-monthly infusions associated to peg-IFN-α and RBV	HCV-related MC cryoglobulinemia with biopsy proven chronic hepatitis	55/ 23/23	5	N	80/0/20	NR	3 mild infusions-related fever episode
Petrarca (2010)	19	PCS	6	-	19	375mg/m^2^ x 4 weekly	Skin Vasculitis 17 Skin Ulcers 3	82/18/0 100/0/0	5	Y	67/33/0	NR	None
Ferri (2011)	87	RCS	6	5	80	375mg/m^2^ x 4 weekly (59 patients) 1 g x 2 administered on days 1 and 15 (18 patients) 375mg/m^2^ x 4 weekly+2 monthly infusions (10 patients)	Skin Vasculits 24 Neuropathy 30 Nephropathy 38 B-NHL 6 Abdominal vasculits 1	62/8/29 50/30/20 50/30/20 50/45/5 100/0/0	29	Not detailed	62/31/7	NR	Infusion-related reactions 4 Infections 4 Mild adverse events 8
Gragnani (2011)	21	RCS	6	-	21	375mg/m^2^ x 4 weekly	Purpura 19 Neuropathy 19 Skin Ulcers 3	68/0/32 58/0/42 67/0/33	5	N	60/0/40	NR	NR
Sneller (2012)	12	RCT	12	-	12	375mg/m^2^ x 4 weekly	MC-Overall Purpura 12 Neuropathy 9 Skin Ulcers 4	50/0/50	4	N	NR	3	1 sever Fever 7 mild adverse effects
Visentini (2011)	27	PCS	12	-	27	250 mg/m^2^ x2 weekly	Overall at 3 months Purpura 24 Neuropathy 23 Skin Ulcers 9	11/59/30 65/30/5 40/45/15 67/23/10	13	N	40/30/30	8	1 serum sickness syndrome, 1 pneumonia, 1 anaphylaxis
De Vita (2012)	28	RCT	24	3	25	1 g x 2 administered on days 1 and 15 (18 patients)	Neuropathy 16 Skin Ulcers 5	12/82/6 80/0/20	7	Y	29/29/42	NR	2 serious infections, 1arterial hypotension followed by angina pectoris
Stasi (2014)	14	PCS	6	-	12	1 g x 2 administered on days 1 and 15 (18 patients)	Purpura 11 Neuropathy 7 Skin Ulcers 1	90/0/10 at 3 months 42/0/68 at 3 months 100/0/0 at 3 months	2	N	50/0/50 at 3 months	NR	NR
Visentini (2015)	52 (evaluable 48)	PCS	12	-	52	250 mg/m2, given twice at one-week interval	Overall Skin ulcers 14 Neuropathy 47 Nephropathy 22	50/31/19	22**	N	NR	NR	6 adverse (1 anaphylaxis)
Roccatello (2016)	31	PCS	72.47 (30-148)	4	26	375mg/m2 × 4 weekly +2 monthly infusions	Overall Skin ulcers 7 Nephropathy 16 Neuropathy 26 Paresthesia Burning feet Weakness	65/32/3 100/0/0 85/5/10 74/16/10 89/5/6	16	Y	75/19/6	9	Drug-related bradycardia N=2 Arterial pressure lowering until 100/60mmHg N=2 UTI N=2

* calculated on those with renal involvement; PCS, prospective cohort study, RCT, randomized controlled trial; CS, cross-sectional, SD, standard deviation; Peg-IFN-α, pegylated interferon- α; RBV, ribavirin; MPGN, membranoproliferative glomerulonephritis; UTI, urinary tract infections; CR, complete response, PR, partial response, NR, no response; N/A, not applicable. **the rate of renal involvement is not detailed in the 48 evaluable patients.

**Figure 2:**
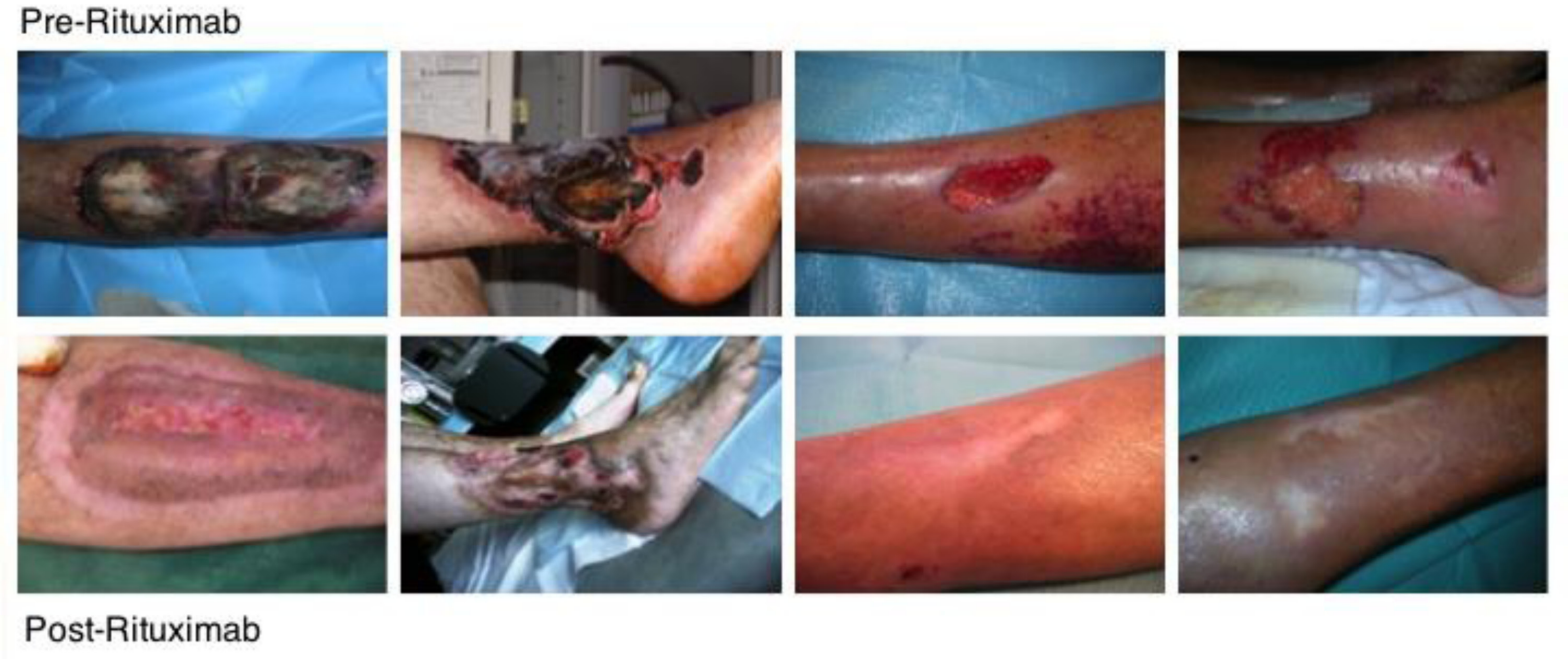
Skin ulcers healing after Rituximab treatment

We recently carried out a very long term prospective (mean follow-up 72.47 months), single-center open study and evaluated the very long-term effects of Rituximab administration in patients with severe cryoglobulinemic vasculitis [46]. Rituximab was given to 31 patients (27 HCV+ve) with mixed cryoglobulinemia (type II in 29 subjects and type III in 2) and diffuse membranoproliferative glomerulonephritis (16 cases), sensitive-motor neuropathy (26 cases) and severe skin ulcers (7 cases). Rituximab was given at a dose of 375 mg/m^2^ (days 1, 8, 15 and 22, followed by 2 more doses 1 and 2 months later, the so-called “4 plus 2 protocol”). Five patients also received 3 pulses of 500 mg of methylprednisolone. No other immunosuppressive or antiviral drugs were added. We found complete remission of pre-treatment active manifestations in all cases of purpuric lesions and non-healing vasculitic ulcers (Figure 2), and in 80% of the peripheral neuropathies. Follow-up after the second month of Rituximab administration revealed a significant improvement in cryoglobulinemic nephropathy, and furthermore, cryoglobulinemic serological parameters, such as low complement C4 and cryocrit also improved. No major side effects were reported. Re-induction with Rituximab was carried out in 9 relapsing patients after a mean of 31.1 months (12-54), resulting again effective. After 6 years of follow-up, the survival rate was 75% and the probability of remaining symptom-free for 10 years without any therapy was approximately 60% after a single “4 plus 2” infusion cycle, while the likelihood of living symptom-free for 5 years after relapse was 80% if treated with the same protocol. Six patients in this study (mean age 75.3 yrs.) died of cardiovascular causes a median of 55 months after their RTX cicle [46]. This open, prospective study showed Rituximab to be safe and effective for treating the most severe cases of MC, even in a very long-term perspective (6 years).

In order to evaluate the available data regarding the use of Rituximab in cryoglobulinemic vasculitis with a particular focus on cryoglobulinemic nephropathy, we searched MEDLINE and EMBASE using the terms “rituximab” and “cryoglobulinemia” in publications between January 1, 1999, to April 1, 2016. Studies were included if they were randomised, controlled studies (RCT) or case series with more than 10 adult patients. Studies were excluded *1*. if they were reviews or expert comments or case series with fewer than 10 patients; *2*. if the main outcome was not clinical, and *3*. if they were published in abstract form alone. When several publications involving the same group of patients were found, only the most recent and comprehensive paper was considered unless the publication was derived from another patient cohort or if the number of included patients was significantly higher.

Among the 233 identified publications, 19 studies met the inclusion criteria, with 16 open-label trials [22–37] and 3 RCTs [38–40]. Selected items were systematically searched for in each paper, i.e., the number of included patients, duration of follow-up, indication for treatment with Rituximab and dosage, concomitant immunosuppressive treatment, corticosteroid dosage and clinical and biological outcomes.

Sixteen open-label trials [22–37], each of which having at least 10 patients with cryoglobulinemia treated with Rituximab, met the inclusion criteria. Data from a total of 440 patients were retrieved. The main indications for treatment with Rituximab were skin involvement (skin vasculitis, purpura and/or skin ulcers) (N=272, 62%), neuropathy (N=254, 58%), and nephropathy (N=143, 33%). Patients received varying doses of Rituximab (2 × 1 g, 4 × 375 mg/m2, 4 +2 × 375 mg/m2 or 2 ×375 mg/m2), as shown in Table 3. Median complete remission (CR) was 68%, partial remission (PR) was 14%, and no response was 10%, respectively.

A further analysis was carried out on case series reporting renal outcomes [23–27, 30, 32–36] (Table 3). These trials included 143 patients, but only a minority had biopsy-proven evidence of cryoglobulinemic glomerulonephritis. Median rates of renal CR and PR and no response to Rituximab in patients with renal involvement in cryoglobulinemia were 57%, 20% and 40%, respectively. Outcomes (overall and renal CR+PR) were defined according to the definitions of each study.

Three RCTs were retrieved [38–40]. In 2010, Dammacco and co-workers [38] investigated the safety and efficacy of a combined use of pegylated interferon-α and ribavirin (RBV), with or without Rituximab, in HCV-related MC. Twenty-two patients with HCV-related MC received pegylated interferon-α (2a: 180 μg or 2b: 1.5μ /kg) weekly plus ribavirin (1,000 or 1,200 mg) daily for 48 weeks, and Rituximab (375 mg/m^2^) once a week for 1 month followed by two 5-monthly infusions [38]. Fifteen additional patients received pegylated interferon-αI and ribavirin with the same modalities, but without Rituximab. CR was achieved in 54.5% (12/22) and in 33.3% (5/15) of patients who received Rituximab and pegylated interferon-α and ribavirin, respectively (p< 0.05), showing that as an add-on to antiviral therapy, Rituximab was well tolerated and more effective than pegylated interferon-α/ ribavirin alone in HCV-related MC.

A prospective RCT investigating the use of Rituximab therapy in patients with severe MC was carried out by De Vita and colleagues [40]. Fifty-nine patients with MC were randomized to the non-Rituximab group (receiving conventional treatment consisting of 1 of the following 3 options: glucocorticoids; azathioprine or (cyclophosphamide; plasmapheresis) or to the Rituximab group receiving 2 infusions of 1 g each and lowering the glucocorticoid dosage when possible, and with a second course of Rituximab at relapse) [40]. Rituximab appeared to be the best therapy for all 3 target-organ manifestations (skin ulcers, active glomerulonephritis, or refractory peripheral neuropathy). The median duration of response to Rituximab was 18 months. The number of patients who achieved the primary endpoint (survival of treatment, defined as the proportion of patients who continued taking their initial therapy), was statistically higher in the Rituximab group at 1 year (64.3% versus 3.5%, p<0.0001), as well as at 2 years (60.7% versus 3.5%, p<0.0001).

In the same year, Sneller and co-workers [39] published the results of a single-center, open label, RCT. Rituximab (375 mg/ m^2^/week for 4 weeks) was compared to the best available therapy in patients affected by HCV-associated cryoglobulinemic vasculitis in whom antiviral was not effective. Sneller's group enrolled 24 patients (12 in each treatment group). Six months after the beginning of the treatment ten patients in the rituximab group (83%) and 1 patient in the control group (8%) were in remission (p< 0.001). The median duration of remission for Rituximab-treated patients who reached the remission within 6 months was 7 months. Moreover, Sneller's group did not observe any adverse effects of Rituximab on HCV viremia or on the liver.

When focusing on the renal clinical outcome, our review of the literature showed a very heterogeneous response to Rituximab. Our recently published study, which represents the largest single-center cohort of patients with MC and renal involvement prospectively treated with Rituximab [41], showed a greater than 90% rate of any response (CR+PR) at the end of follow-up.

We retrieved 11 studies with reported renal outcomes. The median complete response rate was 57% (*vs*. 75% in our cohort). It may be speculated that the “4 plus 2 (*improved*) protocol might intensify the depleting effect of Rituximab on lymphocytes when compared to other Rituximab regimens, thus maintaining more prolonged B-cell depletion and improving clinical outcome [45].

**Figure 3:**
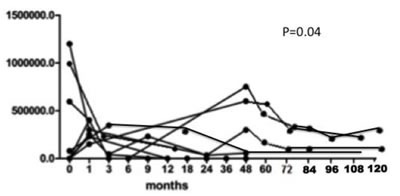
HCV RNA serum load as evaluated at 0, 3, 6, 9, 12 and 18 months and then yearly after Rituximab administration in our cohort of 31 patients with severe mixed cryoglobulinemic vasculitis (type II in 29 cases and type III in 2) with diffuse membranoproliferative glomerulonephritis (#16 cases), peripheral neuropathy (#26) and large skin ulcers (#7)

**Figure 4:**
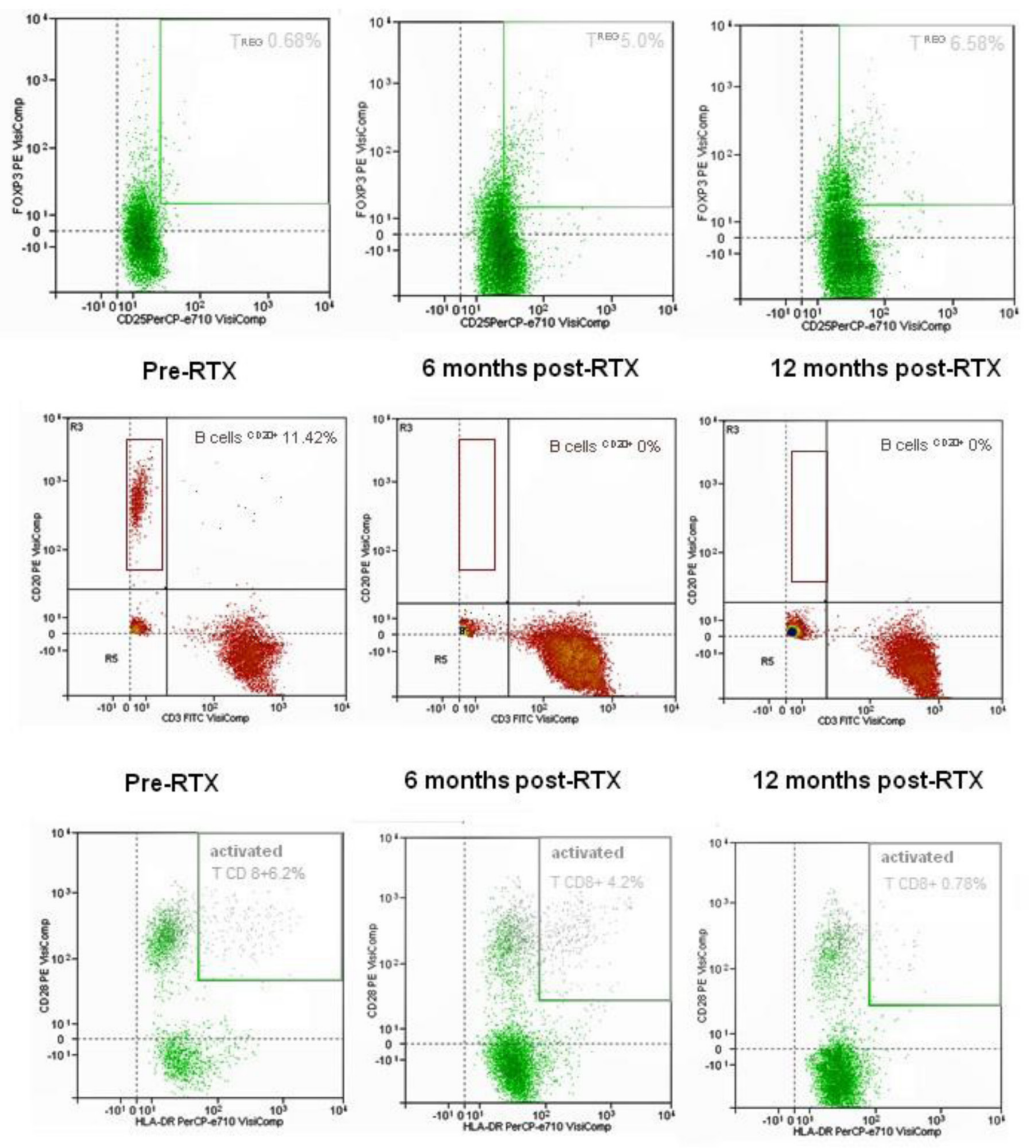
Representative dot-plots of Treg(CD4+CD25+FOXP3+, top plots), B cell (CD 20+, central plots) and activated T CD8+ cell (CD 20+, lower plots) as evaluated by flow cytometry Samples from a responder patient analysed before and at 6 and 12 months after Rituximab administration. Upon detection of B cell depletion, a 9-fold increase in the circulating Treg(CD4+CD25+FOXP3+) and a 7.5-fold decrease in activated T CD8+ cells were observed over 12 months, suggesting Rituximab-induced Th1 cell resetting.

MC is an immune-mediated process that becomes independent from the triggering virus. Rituximab is much more selective than conventional immunosuppressive treatments at interfering with the downstream processes following the disease trigger [41–43], and is definitely safer, as shown in Figure 3 which summarises the data of HCV RNA serum load after Rituximab administration in our cohort of 31 patients with severe mixed cryoglobulinemic vasculitis. Moreover, MC-associated nephritis exemplifies a unique condition of immune-mediated conditions in which Rituximab specifically targets the nephrotoxic Ig-producing cells, potentially inducing a lymphocyte subpopulation re-assessment (Figure 4).

## ANTI-VIRAL THERAPY: THE POTENTIAL ROLE OF DIRECT ANTIVIRAL AGENTS

For more than a decade chronic HCV infection was mainly treated with pegylated interferon (PegIFN) associated with RBV, even though this regimen was poorly tolerated [44]. PegIFN/RBV therapy achieved eradication of infection in fewer than 50% of *naıve* patients with genotype 1 infection [44, 45].

With regard to HCV-associated mixed cryoglobulinemia-associated vasculitis, PegIFN/RBV therapy was found to be most effective when combined with Rituximab. In a pilot study, Saadoun et al. treated 16 consecutive, unselected refractory HCV-MC patients with RTX followed by antiviral therapy with Peg-IFN and RBV. Fifteen out of 16 patients showed clinical improvement with an acceptable safety profile [46]. In 2010, Dammacco et al. and Saadoun et al. published two studies on the use of RTX with Peg-IFN+RBV according to a combined [38] or sequential [30] scheme, respectively. In these and other studies [30, 31, 38, 47], combined therapy with RTX plus Peg-IFN+RBV resulted in better clinical response and higher cryoglobulin clearance than Peg-IFN+RBV alone. This could be due to the combination of cooperating mechanisms: viral eradication and depletion of the pathological B-cell clones. Furthermore, the clinical improvement obtained by adding RTX treatment could make patients who were previously non-eligible for anti-viral therapy now eligible for treatment.

A better understanding of the HCV genome and structures paved the way for the development of direct-acting antiviral agents (DAAs). *Telaprevir* and *boceprevir* were the first DAAs introduced in the clinical practice. Adding these protease inhibitors to PegIFN/RBV in *naıve* patients increased the rate of viral eradication to approximately 70% [48, 49]; however, PegIFN/RBV is still needed since the use of DAAs as monotherapy may cause viral resistance [50, 51]. With regard to HCV-associated mixed cryoglobulinemia with vasculitis, the combination of PegIFNalfa/ribavirin/protease inhibitor proved to be more effective than PegIFNalfa/ribavirin alone [52].

The new *DAAs* showed significant antiviral efficacy (>90% cure) and a good tolerance profile. Years of research have resulted in a very detailed understanding of the viral life-cycle of HCV, leading to new therapeutic strategies. HCV-RNA is bound by the translational machinery of the host cell to the viral internal ribosome entry site. Once internalized, the viral protein is processed into 3 structural and 7 nonstructural (NS) proteins [58]. The new drugs target the 3 nonstructural proteins: NS5A and NS5B RNA polymerase, the NS3 serine protease and its cofactor, NS4A. Telaprevir and Boceprevir represent the first-generation NS3/4A protease inhibitors but were approved for use only in combination with PegIFN/RBV. Asunaprevir, paritaprevir and simeprevir are newly available DAAs. These inhibitors have improved pharmacologic profiles with fewer administrations and better tolerability. Despite the actual role of NS5A is still debated, this structure seems to be involved into the mechanism of viral replication and assembly [59]. Daclatasvir was the first NS5A inhibitor to be launched, [60], followed by ledipasvir [61] and ombitasvir [62]. Overall, these DAAs are effective against a wide spectrum of HCV genotypes [59]. However, those agents have a relatively low resistance threshold. Thus, to minimise the emergence of resistance mutations, their combined use is recommended. Two classes of NS5B inhibitors have been developed: nucleoside and nonnucleoside inhibitors. The nucleoside inhibitors have a particularly high thresholds to resistance due to variants in the active site, which is highly preserved across HCV genotypes, leading to a pan-genotypic activity to DAAs [63]. To date, sofosbuvir is the most advanced DAAs: initially approved to be used in association with RBV for HCV infection (genotypes 2 and 3), it is now the first all-oral, PegIFN-free regimen. Subsequenlty, sofosbuvir was approved for a combined use with simeprevir (NS3/4A serine protease inhibitor) or with ledipasvir (NS5A inhibitor). Beclabuvir and dasabuvir are non-nucleoside inhibitors of NS5B which recognize sites other than the active site and interfere with HCV viral elongation [64–66].

Multidrug regimens are combinations of an NS3/4A inhibitor, an NS5A inhibitor and a non-nucleoside NS5A inhibitor. A full 3D treatment regimen achieves an SVR rate of >95 percent when administered for 12 weeks to naıve patients, and >90 in prior non-responders.

DAAs are expected to modify both the incidence of vasculitis resulting from a prolonged history of HCV infection and the therapeutic algorithms in the early stages of the disease. However, these agents do not possess the immunomodulatory effects of the interferons. They likely do not interfere with the pathogenesis of MC vasculitis nor effectively impact on the development of the immune-mediated injury once the immune disorder is established. Besides, the uncertainty of their pharmacokinetics and safety in the presence of renal impairment requires some caution in their use in nephritic patients.

Limited evidence is currently available investigating the treatment with DAAs in patients with HCV-related MC [52–55], and results are difficult to interpret [56, 57]. Makara[58] described the remission of multiorgan involvement and withdrawal of serum cryoglobulins after initiating DAA therapy as early as 4 weeks in a 40-year old man with severe hepatitis C virus-associated cryoglobulinemia treated for 12 weeks with ombitasvir/paritaprevir/ritonavir, dasabuvir and ribavirin. However, this patient had received 1,600 mg of Rituximab in four doses 5 months earlier. Rituximab probably contributed to the favourable response, in particular on the polyneuropathy which is known to require at least 6-9 months to partially revert [29]. Details on the response profile of proteinuria were not available in that study. Sise at al. [56] showed that at 12 weeks patients with HCV-MC associated vasculitis had sustained virological response rate of 83% (10 out of 12 patients) for sofosbuvir-based DAA regimens. These findings were statistically significantly higher than the historical control groups, which underwent treatment with pegylated interferon and ribavirin. The patients with glomerulonephritis (7 out of 12) who were treated with DAA therapy showed a reduction in proteinuria and, concomitanly, an eGFR improvement. The reduction in proteinuria was particularly evident in the patients with a recent onset of proteinuria. However, by examining the paper in detail, it should be emphasised that during follow-up only 2 patients had a substantial amelioration of serum creatinine (one who was concomitantly treated with Rituximab and one who only had a clinical diagnosis because no biopsy had been performed). Changes were negligible in 4 patients, while serum creatinine increased over time in the remaining patient. About urinary abnormalities, the only patient who had nephrotic range proteinuria at baseline (and showed a decrease to non-nephrotic values) received Rituximab together with the DAAs. Another patient who showed a moderate reduction of proteinuria (from 1,574 to 800 mg/g sCr) was concomitantly treated with ustekinumab, an anti IL-12-23 monoclonal antibody, whose effects in proteinuric patients is presently unknown. Proteinuria decreased from 2,141 to 400 mg/g sCr in a patient who had not undergone biopsy. Of the remaining 4 patients, proteinuria was negative in one case, not determined in another, and only determined by urinalysis (1 and 3+, respectively) in 2 cases.

Sollima et al.[59] treated 7 consecutive patients with HCV-associated mixed cryoglobulinemia vasculitis with new DAAs within an expanded access program. The patients showed a broad range of manifestations, including severe arthralgias, extensive purpura, peripheral neuropathy, skin ulcers and nephropathy. Nephropathy was the most frequent manifestation, presenting as a severe nephrotic syndrome, stages III-IV chronic kidney disease or both. Patients were treated with a variety of IFN-free DAA regimens, including ombitasvir/paritaprevir/ritonavir and dasabuvir, sofosbuvir plus ribavirin, sofosbuvir plus daclatasvir and sofosbuvir plus simeprevir, depending on HCV genotypes. Treatment was given for 12 weeks in five cases and 24 weeks in two. All patients achieved SVR at post-treatment week 12 (confirmed at week 24 in five of them, on a longer follow-up). Serum cryoglobulins were undetectable in 4 patients at the end of treatment but increased again in three during follow-up. At post-treatment week 12, clinical response was observed in only 2 patients. Specifically, the response was partial in one patient and complete in another who experienced vasculitis relapse despite HCV RNA still being undetectable. Thus, SVR to IFN-free antiviral therapy in severe HCV-associated mixed cryoglobulinemia patients may lead to no clinical improvement.

Cornella and coworkers [57] described a case series of 5 patients with genotype 1 chronic HCV-related hepatitis complicated by MC who received 24 weeks’ triple therapy with oral antiviral agents (boceprivir or telaprivir and sofosbuvir). Clearance of serum cryoglobulins was not present in any of these patients.

Notably, a complete remission of MC associated with sustained virological response following a combined Peg-IFN+RBV+DAA (boceprevir)+Rituximab regimen was described by Urraro et al.[60].

Taken together these observations emphasise the role of DAAs in eradicating HCV infection even in mixed cryoglobulinemia patients, a subset that is less responsive to conventional antiviral treatment with Peg-IFN/RBV. However, more studies are needed to establish the ideal protocol and duration of therapy with DAAs for chronic HCV and coincident mixed cryoglobulinemia.

Meanwhile, interest in Rituximab therapy in the most severe patients with mixed cryoglobulinemia remains unchanged. Besides its immunomodulatory effect, Rituximab also plays an important role by depleting CD19 positive-B cells, known to be HCV reservoirs. Improving the effects on vasculitis treatment and HCV eradication using a combination of DAAs and Rituximab can be envisaged.

## THERAPIES TARGETING ALTERNATIVE PATHWAYS

The use of other drugs, including thalidomide [61, 62], lenalinomide [63], and bortezomib-based regimens [64, 65], may be useful in patients with type I cryoglobulinemia. Some anecdotal case reports describe successful approaches with abatacept [41] or tocilizumab [66]. B-cell activating factor (BAFF) blocking agents [67] and interleukin-2 agonists [68] may be promising future therapies. Again, the combination of these agents with DAAs could lead to parallel control of the vasculitic process and eradication of the viral trigger.
